# New York Odontological Society

**Published:** 1886-02

**Authors:** 


### NEW YORK ODONTOLOGICAL SOCIETY.

Reported Expressly for the Independent Practitioner.

A regular meeting of this society was held in the parlors of the Academy of Medicine, Tuesday evening, Jan. 12th, the President, Dr. J. Morgan Howe, in the chair.

Under the heading "Incidents of Office Practice," Dr. Chas. Miller presented casts of a regulating case, showing a decidedly marked improvement under his treatment, also exhibiting the appliance by which the result was obtained. The patient whose teeth the cast represented was a young lady whose inferior incisors projected far outside the superior teeth. The first inferior bicuspids were at once removed, and appliances of black vulcanized rubber were made to cover the inferior molars on either side, to which were attached rubber rings, and to these a platinum bar was tied, which bar extended from one ring to the other outside the inferior teeth, thus pressing against the teeth and contracting the arch until the teeth were moved to the desired position.

*Dr. W. H. Dwinelle*—Among other interesting cases described, referred to a superior central incisor which he treated some fifteen years ago. From a loss of neighboring teeth this incisor has done extra service and been subjected to a severe strain, consequently was badly worn away, nearly to the pulp chamber. Wishing to preserve its vitality and make it useful, he inserted small screws on either side of the pulp for the purpose of obtaining anchorage, and then built out the point with gold. Recently the tooth became devitalized and broke away, so was extracted, but the bright burnished surface of the gold point gave evidence that it had sustained much hard use. The doctor exhibited a superior molar, one of the roots of which had been excised, yet nature had accommodated herself to this condition so that the organ had been retained in the mouth to do good service for years. He also presented an incisor with two roots which, with other teeth in his possession, he found among a lot of bones exhumed from the site of the old New York postoffice, which had been an ancient churchyard.

*Dr. F. H. Wardwell*—Stated his experience in capping exposed pulps, and related the number of times he had capped and filled, then recapped and refilled; one case in particular in which the stopping of oxy-phosphate of zinc had from time to time worn away, and been repeatedly renewed during a number of years.

*Dr. F. Y. Clark*—Presented a tooth showing exostosis where there had been an occluding tooth, stating that he believed Dr. Atkinson had offered a "Delmonico dinner" to any one who could show such a condition in a tooth having an articulation.

*Dr. W. A. Bronson*—Read a short paper, taking for his subject: "Dead Teeth and their Treatment." He stated that there probably was nothing new that he could suggest differing materially from the treatment adopted by many others, but he had been requested by Dr. Woodward to give his usual methods of treating dead teeth, which he did. The paper was freely discussed, and the advisability of drilling through the alveolus to reach the apex of a root was a disputed point at issue.

*Dr. Dwinelle*—Had, in some cases, drilled through the alveolus, but did not make this his usual practice.

*Dr. E. T. Payne*—Stated that he performed this operation, and did not consider it a painful one if skillfully and carefully done. He made a small incision in the gum with a lancet, then taking a small bur, ground on a stone to a spade shape, placed it in the engine and passed it so rapidly and easily through the alveolus that the patient would hardly be aware of it.

*Dr. Clark*—Thought this method of treatment barbarous. He is able to treat his cases of dead teeth by openings through the nerve canals, believing these openings nature's guide to the seat of trouble.

*Dr. W. T. Laroche*—Asked Dr. Clark how, in cases where the membrane surrounding the root had become thickened, inflamed, and in a sloughing condition, he could relieve and get rid of the trouble without opening through the alveolus?

*Dr. Clark*—Replied that if the nerve canals had already been filled and the trouble occurred afterwards, he might possibly resort to the method referred to, for when he filled roots it would be impossible to remove his fillings, but he was careful to treat the root canals and fill temporarily, and allow them to so remain a long time before inserting permanent stoppings.

*Dr. Laroche*—Stated that he made free openings in these cases, and enlarged the space by means of a cotton tent, then removed the diseased portion of the membrane and scraped the ends of the affected roots. He did not think he had any failures.

*Dr. Clark*—Asked if the incision healed kindly and closed en-entirely; to which Dr. Laroche gave an affirmative reply.

*President Howe*—Intimated that this opening through the alveolus was considered proper surgical treatment in such cases.

*Dr. J. W. Clowes*—Related an instance where an old gentleman consulted him in regard to a troublesome molar, though he had just been in the hands (as the doctor expressed it) of a "high-toned dentist" who had pronounced his teeth all right. On examination, Dr. Clowes discovered the tooth to be very sensitive, owing to an encroachment upon the pulp from a wearing down of the crown. As the "high-toned dentist" had stated that he could do nothing more to the tooth, Dr. C. concluded to try. He formed a pocket by drilling into the tooth, placing therein a devitalizing paste, and when dead, the nerve was extracted and the canals and cavity filled, thus giving to the patient peace and comfort.

*Dr. B. Lord*—Read a brief paper on the extraction of teeth, in which he gave it as his opinion that, if possible, the first permanent molars should always be saved, they being in position to do much of the masticating work. He thought that even where the crowns of these teeth were broken down, it was best to fill the nerve canals and retain the roots in position so long as they gave no trouble, thus preventing the neighboring teeth from falling towards each other, or becoming sensitive at their necks from absorption of the alveolus.

*Dr. J. Smith Dodge, Jr.*—Remarked that as one gentleman would always extract the first bicuspids to secure space, another would always remove the second bicuspids, and another would always select the sixth-year molars for the sacrifice, there seemed no rule except as each made one for himself. He himself was in the habit of extracting such teeth as ought to come out, and he did not believe that any fixed rule could be carried out, as different patients presented conditions of a different character. In some mouths the molars might all be good and the bicuspids all bad, or vice versa. He was of the opinion that the statement which had been made "that it was the dentist's place to save all the teeth," was wrong, for often by removing certain teeth, even though sound, it would leave the other teeth in the mouth in a much better condition, and they would be longer retained.

*Dr. B. F. Howland*—Read a short paper on "Tooth Crowns," also described an invention of his own. He presented a specimen to the society for examination, and hoped it might be of benefit to dentistry. He did not wish to disparage the efforts of others who had invented crowns, for many had done excellent work, but many crowns in use had weak points, and he had endeavored to produce something which might overcome such objectionable features.

*Dr. Davenport*—Spoke highly of the "Howland Crown," he having made use of it in a number of cases. They were simple, exceedingly easy of adjustment, and when in position presented a much more natural appearance, both inside as well as on the surface exposed, than any he had used.

Dr. J. B. Littig, and Dr. Dwinelle both commended this crown for the above mentioned good points.

*Dr. Dodge*—Had very good results in using the old pivot crowns, substituting in place of the wooden dowel a piece of heavy gold wire, roughening the end and first fastening it into the tooth with cement, then when it was set, fastening the other end of wire in the root with cement, the crown then being attached.

